# CD20+ T Cells in Multiple Sclerosis: From Pathogenesis to Treatment-Induced Depletion

**DOI:** 10.3390/ijms26146655

**Published:** 2025-07-11

**Authors:** Anna Chiara Mazzeo, Laura Calabresi, Valentina Damato, Gregorio Spagni, Luca Massacesi, Alice Mariottini

**Affiliations:** 1 Department of Neurosciences, Psychology, Drug Research and Child Health (NEUROFARBA), University of Florence, 50139 Florence, Italy; annachiara.mazzeo@unifi.it (A.C.M.); laura.calabresi@unifi.it (L.C.); valentina.damato@unifi.it (V.D.); gregorio.spagni@unifi.it (G.S.); 2 Department of Emergency Neurology, Careggi University Hospital, 50139 Florence, Italy

**Keywords:** multiple sclerosis, encephalomyelitis, autoimmune, experimental, antigens, CD20, T-Lymphocyte Subsets, monoclonal antibody, autoimmune diseases

## Abstract

The traditional paradigm of multiple sclerosis (MS) as a T cell-mediated disorder has been challenged by the effectiveness of monoclonal antibodies (mAbs) targeting CD20-expressing lymphocytes. Although these are mostly represented by B cells, the CD20 marker is expressed by 2–6% of T cells (CD20+ T), which are effectively depleted in serum and cerebrospinal fluid of MS patients by anti-CD20 mAbs. CD20+ T cells are characterized by a pro-inflammatory phenotype and increased potential for migrating and invading the central nervous system (CNS) compared to CD20− T cells. Furthermore, CD20+ T cells are detected within brain inflammatory lesions from MS patients and actively participate in the experimental MS model. This review aims to summarize the current knowledge on CD20+ T cells, from their identification and characterization to evidence of depletion by disease-modifying treatments (DMTs), likely contributing to therapeutic efficacy. Conflicting hypotheses on the origin and development of CD20+ T cells will also be discussed, as well as evidence from clinical and preclinical studies supporting their pathogenetic role in MS.

## 1. Introduction

Multiple sclerosis (MS) is a chronic inflammatory demyelinating and degenerative disease of the central nervous system (CNS) that has long been considered as mediated by T lymphocytes [1]. However, over the past decades, this paradigm has been challenged by the remarkable effectiveness of therapeutic monoclonal antibodies (mAbs) depleting B cells through the engagement of the surface marker CD20 [2,3]. In addition to the antibody production, pathogenetic roles of B cells likely include promotion of T cell activation by acting as antigen-presenting cells (APCs) and production of pro-inflammatory cytokines, which contribute to tissue damage and demyelination in both white matter (WM) and deep/cortical grey matter (GM). Interestingly, although historically considered a specific B cell marker, CD20 is also expressed by a small percentage of T lymphocytes, although at low levels (CD20_dim_ or CD20+ T cells) [4,5]. CD20+ T cells show an activated proinflammatory phenotype and have been increasingly implicated in the pathogenesis of autoimmune diseases, particularly MS and its animal model, experimental autoimmune encephalomyelitis (EAE). CD20+ T cells are effectively targeted by anti-CD20 mAbs, raising the intriguing hypothesis that their depletion may contribute to the therapeutic efficacy of such molecules [6,7,8].

In this narrative review, after an overview of the potential origin of CD20+ T lymphocytes, the evidence supporting their role in MS pathogenesis is summarized from preclinical data on EAE and clinical research. Studies focusing on the effect of available disease-modifying treatments (DMTs) on CD20+ T cells are also reviewed, highlighting the reported associations between depletion of this subpopulation and treatment effectiveness.

## 2. Materials and Methods

PubMed was searched for studies written in the English language and published up to 31 December 2024, focusing on the following topics: a description of CD20+ T cells in healthy donors (HD), MS, and EAE; the effect of DMTs on CD20+ T cells in MS. The keywords used for the search included the MeSH terms “Multiple Sclerosis”, “Encephalomyelitis, Autoimmune, Experimental”, “Antigens, CD20”, and “T-Lymphocyte Subsets”. Papers were searched independently by two authors, resulting in a total of 27 studies included.

## 3. Origin of CD20+ T Cells

**Figure 1:**
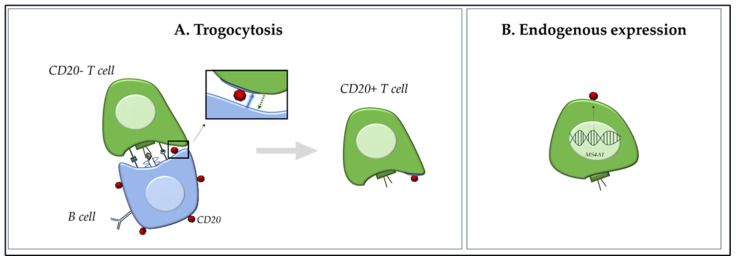
Dual Origin of CD20+ T Cells. Schematic overview of the two proposed hypotheses on the origin of CD20+ T cells. (**A**): Trogocytosis pathway—CD20− T cells acquire membrane-bound CD20 through direct contact with CD20+ B cells during immune synapse formation (blue arrow). This interaction leads to temporary CD20 acquisition without transcriptional activation of *MS4A1* (CD20 gene). The transfer of surface molecules could occur between cells in both ways (green arrow). (**B**): Endogenous expression model—a subset of T cells intrinsically expresses CD20 via transcription of the *MS4A1* gene, indicating the existence of a distinct CD20+ T cell lineage. These cells may maintain stable CD20 expression and can generate daughter cells with a similar phenotype.

CD20, a membrane-embedded phosphoprotein encoded by the *MS4A1* gene, is a well-established B cell marker traditionally associated with B cell development and function. However, the discovery of CD20 expression on T cells has raised intriguing questions on its origin and functional role [4]. Although CD20+ T cells have been observed in peripheral blood and tissues from patients with different diseases, including cancer and autoimmune disorders, the mechanisms underlying their presence and their potential pathogenic implications remain poorly understood. The origin of CD20+ T cells has been debated, with two prominent hypotheses: (1) the acquisition of CD20 from B cells via trogocytosis, and (2) the endogenous expression of CD20 as a marker of a distinct T cell lineage (Figure 1) [9]. Each hypothesis carries significant implications for understanding immune regulation, tumor immunology, and therapeutic targeting, particularly in the context of anti-CD20 mAb therapy. Both these hypotheses are critically examined in the following paragraphs.

### 3.1. Trogocytosis: Acquired Expression of CD20 via Intercellular Transfer

Trogocytosis is a process whereby membrane fragments are transferred from one cell to another through receptor–ligand interactions during direct cell–cell contact [10]. Initially described as a T-cell receptor (TCR)-dependent phenomenon, it is now recognized as a broader mechanism involving various immune and non-immune cell types, including stromal and even tumor cells. In the immune context, trogocytosis would enable the acquisition of membrane components from APCs, such as B cells, through direct cell-cell interactions during immune synapse formation [10]. Several studies demonstrated that CD20− T cells can acquire the CD20 marker when co-cultured with CD20+ B cells in vitro, even in the absence of de novo *MS4A1* mRNA expression, suggesting that trogocytosis is a plausible mechanism for the transient appearance of CD20 on T cells [6,9,11]. This process is highly dynamic and occurs rapidly, often within an hour of cell contact; importantly, it was shown that this process was abrogated when cell-cell contact was prevented by transwell membranes, underscoring that trogocytosis is contact-dependent. These acquired CD20 molecules were often clustered in patches and lacked internalization or sustained signalling, supporting the notion of passive acquisition [11]. Schuh et al. [5] used imaging flow cytometry to distinguish trogocytosed CD20 molecules from endogenously expressed forms. They observed that CD20 on T cells was typically membrane-bound and co-expressed with other B cell markers such as CD19 and CD79a, and that CD20+ T cells lacked transcriptional activity for *MS4A1*. This led to the interpretation that CD20+ T cells in such contexts may be artifacts of B cell contact, particularly in inflamed lymphoid tissues.

However, critics of these models point out that CD20 expression on T cells can be stable for prolonged periods, both in vitro and in vivo, whereas trogocytosed proteins are typically expressed only transiently. Moreover, CD20+ T cells have been identified in the absence of B cells in specific immunodeficient models and following anti-CD20 mAb therapy (e.g., rituximab—RTX), where CD20+ T cells persist despite an extensive depletion of B cells [12].

### 3.2. Distinct T Cell Lineage: Evidence of Intrinsic CD20 Expression

An alternative and increasingly explored hypothesis is that CD20+ T cells represent a distinct T cell subset with lineage-specific expression of CD20, either arising from unique developmental trajectories or acquired during activation [5]. Studies using transcriptional profiling and single-cell RNA sequencing revealed a subset of T cells with endogenous *MS4A1* (CD20 gene) expression, particularly in the tissue-resident memory (T_RM_) and regulatory T cell compartments [7,13]. This suggests that CD20 expression in T cells may not merely be an artifact of surface protein acquisition, but rather a functional marker of a specialized T cell lineage. Palanichamy et al. [7] were among the first to identify CD3+CD20+ T cells in the peripheral blood and cerebrospinal fluid (CSF) of MS patients, describing their enhanced production of pro-inflammatory cytokines (e.g., IFN-γ and IL-17) and their depletion following anti-CD20 therapy (RTX), thereby implicating a potential pathogenic role. These cells were not only phenotypically distinct but also persisted over time, opposite to the transient nature expected from trogocytosis. Expanding on this, von Essen et al. [13] characterized CD20+ T cells in various autoimmune settings. Their analysis revealed that these cells exhibited a memory phenotype (CD45RO+CD27+) and increased activation markers, such as CD69 and HLA-DR. Their transcriptomic profile partially overlapped with T_RM_ T cells, supporting the idea of a functionally specialized subset, rather than a byproduct of B cell interaction. More recently, von Essen et al. [9] provided critical support for the intrinsic expression model using bulk and single-cell RNA sequencing. They identified a subset of CD4+ and CD8+ T cells that actively transcribe *MS4A1*, exhibit chromatin accessibility at the *MS4A1* locus, and express other B cell-like genes in a regulated fashion; moreover, they identified a subset of naïve T CD20+ cells (CD31, CD62L, and CD27^hi^CD38^hi^), further supporting the hypothesis of an alternative developmental pathway compared to trogocytosis. Importantly, CD20+ T cells were enriched in inflamed tissue sites such as CNS lesions in MS and synovial fluid in rheumatoid arthritis, further suggesting their functional role in inflammation. In cancer immunology, De Bruyn et al. [11] identified CD20+ T cells in ascitic fluid of patients with ovarian cancer.

### 3.3. Reconciling the Models: Complementary Mechanisms?

It is indeed plausible that both these mechanisms contribute to the generation of CD20+ T cells under different circumstances. While trogocytosis may explain a short-lived expression of CD20 associated with cell activation in immunological synapses, particularly during high B–T cell interactions in secondary lymphoid organs, the lineage model better accounts for the stable, functionally distinct CD20+ T cell population observed in peripheral blood and sites of active inflammation. Thus, rather than being mutually exclusive, trogocytosis and transcriptional regulation may coexist, reflecting different immune states or microenvironmental cues. Further studies employing single-cell transcriptomics, epigenetic profiling, and lineage tracing in model systems are necessary to fully resolve the origin and immunological role of CD20+ T cells.

## 4. Features of CD20+ T Cells

The first description of CD20+ T cells in humans dates back, to our knowledge, to 1993, when two distinct populations of CD20-reactive lymphocytes, previously described using commercial CD20 mAb reagents in routine immunophenotyping [14,15], were characterized in HD by demonstrating that the CD20 marker was expressed at either high or low density, hence defined as CD20_bright_ and CD20_dim_ population, respectively. Whereas the former clearly represented B cells, as it expressed the surface molecules Ig and CD19, the latter was mostly constituted by a subpopulation of T cells [4]. This T cell population was, indeed, similar to other CD20− T cells, with the same expression of CD2, CD5, CD56, CD57, and HLA-DR [4], but higher expression of CD8 than CD4 [6,16]. As a consequence, the CD4:CD8 ratio was reduced in the CD20+ T compared to the CD20− T compartment in HD (0.6 vs. 1.8, respectively) [4], as well as in the CD3+ pool in both a relapsing-remitting (RR-)MS cohort (1.4 vs. 2.5) and HD [13]. Similar findings were reported in another MS cohort, where CD20+CD3+ cells represented 18.4 ± 2.3% of all CD20+ cells and included a higher proportion of CD8+ (58.9 ± 2.6%) than CD4+ (35.1 ± 2.4%) cells, opposite to the entire CD3+ T cell compartment (29.2 ± 2.4% vs. 69.3 ± 2.4%, respectively) [17]. Accordingly, the mean CD20 fluorescence intensity (MFI) was higher on CD8+ than CD4+, but, obviously, always lower compared to CD20+CD19+ cells (MFI: 40,262 ± 3208) [17]. Partially aligned with these findings, co-expression of CD4 and CD8 was observed in 54% (±20%) and 46% (±17%) of CD20+ T cells in the study by Konen et al., including 53 relapsing MS patients, where T cells represented 13% (±6%) of the CD20+ lymphocytes [18].

Frequencies of CD20+ T cells in HD range from 2% to 6% of T cells across different studies, with partially conflicting data on whether their frequency differs between MS and HD [4,5,7,12,19,20,21]. Compared to HD, Holley et al. [19] reported a similar frequency of CD3+CD20+ cells in MS, whereas Von Essen et al. [13] described an increased frequency in both the CD4+ and CD8+ compartments of RR- and primary-progressive (PP-)MS. CD20+ T cells frequency shows a remarkable interindividual variation with individual levels stable over time [4], and it is generally reported to be not age-related [20], except for one study by Storie et al., which describes a significant correlation with age, with CD20+CD3+ T cell frequency at 0.3 ± 0.1% in the cord blood, growing thereafter to 2.1 ± 1.1% in the 20-60-year-old group, and up to 6.9 ± 3.2% in the >60-year-old cohort [21].

CD20+CD3+ cells isolated from both HD and MS patients are a heterogeneous T cell population exhibiting predominantly central and effector memory (T_EM_) phenotypes, with enhanced activation and production of proinflammatory cytokines [6,7]. CD20+ T cells show features of higher differentiation compared to CD20− T cells, with lower proportions of naïve and stem cell memory cells, higher proportions of T_EM_ cells and terminally differentiated cells [13,16], and a pro-inflammatory phenotype (higher expression of CD49d, especially on CD4+, IFN-γ, and other pro-inflammatory cytokines) [19]. More recently, these results were confirmed in a cohort of 17 HD [22], where CD20+ T cells presented a higher proportion of transitional memory cells compared with CD20− T cells and displayed a proinflammatory phenotype characterized by the expansion of T helper (Th)1, Th1/17, and T cytotoxic (Tc)1 cell subsets, associated with a high expression of activation (CD25) and exhaustion (PD-1) markers.

Upon stimulation with phorbol 12-myristate 13-acetate (PMA) and ionomycin, CD20+ T cells from HD produced IL-4, IL-17, TNF-α, and IFN-γ more readily than CD20− T cells [5,13]; in the sub-analyses, CD4+CD20+ T cells produced more IL-4, IL-17, and TNF-α than their CD8+ counterpart, whereas CD45RO+CD20+ T cells produced more TNF-α than the CD45RA+ counterpart [5]. A similar pattern of cytokine production was reported in HD by Pinho et al.; in this study, the authors also identified, for the first time, CD4+CD20+ T cells simultaneously producing the proinflammatory cytokines IFN-γ, TNF-α, and IL-17 [22]. These patterns are consistent with a Th1 and Tc1 phenotype for CD4+ and CD8+ CD20+ T cells, respectively, as confirmed by the description of a higher frequency of Tbet-expressing CD20+ T cells compared to CD20− T cells, significant for both the CD4+ and CD8+ subpopulations [11,13].

RNA sequencing of CD20+ T cells from HD revealed upregulation of genes associated with enhanced inflammatory phenotype (BAG6, CARD16, IFIT2, PARP12, and eIF3i), differentiation into memory CD8+ T cells (SMAD4, E2F4, and MAP3K5), and enhanced blood-brain barrier (BBB) adhesion/migration (GZMK, Integrin alpha 5, LMO7, and SELL), with downregulation of inhibitors of cell adhesive properties (ICAP-1, CANX, and CMTM3) [23]. An example of how this peculiar gene expression pattern affects the distribution of CD20+ T cells is offered by an experiment in RAG2-/-γc-/- mice [23]. In animals sacrificed after the in vivo injection of primary human T cells transduced with a CD20-encoding retroviral vector (25% transduction efficiency) or fresh human peripheral blood mononuclear cells (PBMCs, used as control), wild-type CD3+ T cells were detected as fully dispersed throughout the spleen, whereas almost all the CD20+CD3+ T cells showed a characteristic localization in the splenic periarteriolar lymphoid sheath, with a distribution similar to that of B cells in control mice. This observation suggests that CD20 expression alters T cell homing ability.

## 5. CD20+ T Cells and MS Pathogenesis

Immune cells, both innate and adaptive, and CNS resident cells are all involved in MS pathogenesis, with heterogeneous roles across different phases of the disease [24]. Briefly, early stages are characterized by high levels of new focal inflammatory activity (relapses and new magnetic resonance imaging—MRI—lesions in the brain and/or spinal cord), and MS pathogenesis is driven by the formation of inflammatory infiltrates within perivenular spaces (also known as Virchow-Robin spaces) of brain and/or spinal cord WM. These infiltrates, named as perivascular cuffs, are mainly composed of autoreactive T lymphocytes (mostly CD4+) which, after being activated in the periphery by the encounter with APCs expressing their cognate antigen, cross the BBB and release pro-inflammatory cytokines, promoting further immune cell recruitment. Damage and demyelination to the WM surrounding the perivascular cuffs are promoted by diffusion of neurotoxic cytokines, cell-to-cell interactions, and complement activation; brain parenchyma is infiltrated by pathogenetic T CD4+ and CD8+ cell clones, sparse B lymphocytes, and plasma cells. In this environment, innate immune cells, including macrophages and resident microglia, play a dualistic role, acting as scavengers of cellular debris, a step critical for subsequent remyelination and tissue repair, and contributors to tissue damage through the release of pro-inflammatory cytokines and damage-associated molecular patterns (DAMPs) [25].

In the chronic phase, inflammatory infiltrates within the parenchyma are less prominent, and acute lesions gradually evolve towards chronic active or inactive lesions [26]. The former, also defined as smoldering lesions, are characterized by a rim of iron-laden macrophages/microglia at the lesion’s edges and gradually expand towards the surrounding normal-appearing (NA)WM [27]. Smoldering lesions are considered a hallmark of compartmentalized inflammation, along with leptomeningeal infiltrates of B cells, CD8+ T cells, and dendritic cells, which may aggregate, resembling tertiary lymphoid organs (i.e., follicle-like structures) [28].

Evidence from studies on EAE and MS supporting an active role of CD20+ T cells in MS pathogenesis is summarized in the following sections. Studies describing the main features of CD20+ T cells isolated from peripheral blood, CSF, and the brain of HD or MS patients are summarized in Table 1.

### 5.1. Role of CD20+ T Cells in EAE

In the seminal study by Ochs et al. [6], CD20+ T cells were characterized in the context of EAE, providing compelling evidence that this subset plays a non-redundant and pathogenic role in disease development. The authors adopted both wild-type and transgenic mouse models with conditional depletion of CD20+ cells to dissect the contribution of CD20-expressing T cells versus B cells. They demonstrated that the depletion of CD20+ cells significantly ameliorated clinical scores in EAE and, importantly, selective depletion of CD20+ T cells without affecting B cells significantly reduced demyelination and tissue infiltration by CD3+ cells compared to control-treated mice. This finding underscored the functional independence and pathogenic potential of CD20+ T cells in CNS autoimmunity.

Phenotypically, CD20+ T cells represent a subset of primarily memory/effector CD8+ and CD4+ T cells with high pro-inflammatory potential. Ochs et al. showed that these cells expressed elevated levels of IFN-γ, GM-CSF, and TNF-α, all cytokines known to mediate neuroinflammation in EAE [6]. Furthermore, CD20+ T cells exhibited enhanced CNS-homing properties, with increased expression of integrins and chemokine receptors such as VLA-4 (α4β1 integrin) and CXCR3, which facilitate the migration across the BBB. Another striking feature was their potential to act as APCs: through the expression of MHC class II and co-stimulatory molecules (CD80/CD86), CD20+ T cells were able to activate other T cells, suggesting their possible contribution to epitope spreading and sustained autoimmunity [6]. This APC-like function may partially explain the robust therapeutic response to anti-CD20 mAbs observed in MS patients, which likely stems from the simultaneous targeting of pathogenic B cells and CD20+ T cells.

Ochs et al. also addressed the temporal dynamics of CD20+ T cell infiltration in the CNS, observing that this occurs early in EAE development, when these cells are detected particularly in the perivascular cuffs and meninges. Their early presence may prime the local immune microenvironment and facilitate subsequent infiltration by other effector cells, further exacerbating demyelination and neurodegeneration.

**Figure 2:**
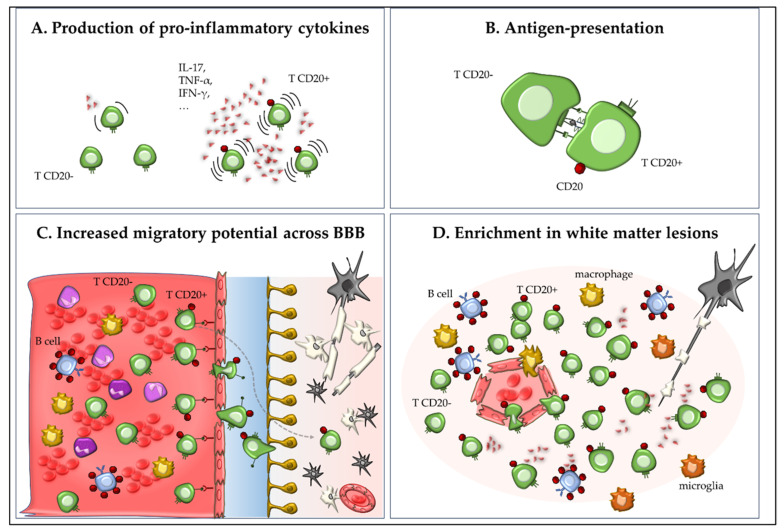
Role of CD20+ T cells in experimental autoimmune encephalomyelitis (EAE) and multiple sclerosis (MS). CD20+ T cells represent a small subset of memory/effector CD8+ and CD4+ T cells with high proinflammatory capacity. In EAE and MS, these cells produce pro-inflammatory cytokines such as IL-17, IFN-γ, GM-CSF, and TNF-α (**A**); may act as antigen-presenting cells through expression of MHC class II and co-stimulatory molecules (experimental data from EAE), thereby contributing to neuroinflammation and autoimmune pathology (**B**); exhibit enhanced migration into the CNS via expression of integrins (e.g., VLA-4) and chemokine receptors (e.g., CXCR3; (**C**)); and are enriched in inflammatory CNS lesions (**D**).

In summary, CD20+ T cells represent a distinct, pro-inflammatory T cell subset with critical involvement in the pathogenesis of EAE. Their ability to produce pro-inflammatory cytokines, present antigens, and migrate to the CNS is consistent with key mechanisms known to drive autoimmune neuroinflammation (Figure 2). The findings by Ochs et al. thus broaden our understanding of CD20-targeted therapies, suggesting that therapeutic efficacy may derive not only from B cell depletion but also from the removal of pathogenic CD20+ T cells [6].

### 5.2. Role of CD20+ T Cells in MS

#### 5.2.1. Pro-Inflammatory Phenotype and CNS Migratory Potential

A putative pathogenetic role of CD20+ T cells in MS is suggested by the demonstration of their enhanced activation and pro-inflammatory phenotype, as summarized in the previous section, “Features of CD20+ T cells”. Further evidence on this matter is provided by the observation of a more vigorous pro-inflammatory response of CD4+CD20+ T cells compared to CD20− T cells to the CNS antigens myelin oligodendrocyte glycoprotein (MOG) and myelin basic protein (MBP), with no difference in CD20+ T cells reactivity between RR-MS patients and HD, except for a non-significant trend to an increased MBP reactivity in RR-MS (13.6% vs. 6.6%) [13]. CD20+ T cells from MS patients and HD also had increased proliferative capacity and expression of the apoptotic markers annexin V, Fas, and FasL compared to CD20− T cells [13].

In the study by Sabatino et al., MS patients showed a significantly higher frequency of CD20+CD8+ cells specific to pooled myelin antigens (10.1 ± 2.7% vs. 3.3 ± 1.1%, *p* = 0.01) and MOG_181–189_ peptide (21.2 ± 7.8% vs. 0%, *p* = 0.0002) compared to HD, associated with an overall significant increase in myelin-specific memory CD20+CD8+ T cells (53.7 ± 10.3% vs. 27.0 ± 9.7%, *p* < 0.05) [8]. This latter observation is consistent with an increased activation state in MS and supports the notion that a greater proportion of myelin-specific CD8+ T cells have encountered such antigens in MS patients compared to HD [8].

In addition to the pro-inflammatory phenotype and higher response towards myelin antigens, a higher CNS migratory potential was described in CD20+ T cells isolated from both peripheral blood and CSF. In detail, von Essen et al. reported a significantly increased expression of chemokine receptors (CCR2, CCR5, CCR6, CXCR3) and adhesion molecules such as CD49d and MCAM-1 (significant in the CD4+ population only) in the CD20+ compared to the CD20− compartment of T cells isolated from the peripheral blood of RR-MS patients; interestingly, no differences in the expression of such molecules were found in CD20+ T cells from MS and HD, suggesting that higher CNS migration potential in T cells is deeply associated with the expression of the CD20 marker [13]. Amongst chemokines, CCL-3 was substantially increased, determining an increased adhesion of CD20+ T cells to endothelial cells of human brain microvessels, possibly enhancing CNS inflammation through the recruitment of immune cells from the peripheral blood [13]. The great migratory potential of peripheral CD20+ T cells from RR-MS patients was substantiated by the observation that this population was enriched in the CSF, where it showed a higher expression of migration molecules (such as CCR2 and CCR5 on both CD4+ and CD8+; MCAM-1 and CCR6 on CD4+ only) compared to CD20− T cells [13].

#### 5.2.2. Enrichment in the Brain and CSF

Immunohistochemistry studies on post-mortem human brains from HD and people with different disorders (including MS and CNS degenerative diseases) revealed a predominant presence of CD8+ T_RM_ cells [29,30], with enrichment of CD8+CD69+CD20+ T_RM_ compared to peripheral blood [31]. These findings were replicated in MS brains through quantitative reverse transcription-polymerase chain reaction (RT-PCR) assay, showing that mRNA encoding CD20 (*MS4A1*) is constitutively expressed by brain CD8+ T_RM_ cells [31]. CD20+CD3+ cells, irrespective of their isolation from WM of HD, NAWM, and WM lesions of MS patients, showed a high expression of markers for tissue homing (CXCR6), proliferation (Ki-67), and cytotoxicity (granzyme B) when compared to CD20-CD8+ T_RM_ cells, regardless of CD103 expression [31]. This is consistent with the detection of CD4+ and CD8+ CD20+ T cells in acute and chronic WM lesions of MS, but not in post-mortem WM of HD [19]. Analyzing the perivascular space surrounding active WM lesions, Hsiao et al. confirmed the presence of a pro-inflammatory environment consisting of CD20_bright_ B cells and CD20-CD3+ and CD20_dim_ cells, detected in higher proportion compared to control WM [31].

Differences in the frequency of CD20+ T cells in the CSF were inconsistently observed across studies comparing MS patients with different controls. Indeed, CD20+ T cell frequency, both in the CSF and peripheral blood, was similar between eight patients with other non-inflammatory neurological disorders (OND, defined by the absence of all the following: pleocytosis, disturbance of BBB permeability, and intrathecal IgG production) and six MS patients on relapse [5]. Conversely, the CSF was enriched with CD20+ T cells (in both the CD4 and CD8 compartments) compared to the peripheral blood in 25 RR-MS [13] and 25 untreated PP-MS patients; these results were validated in a replication cohort including 16 untreated PP-MS [32].

Furthermore, a clue on the pathogenetic role of CD20+ T cells derives from the positive correlation observed between their frequency in the CSF and both CSF MBP concentration and Expanded Disability Status Scale (EDSS) scores [13]. Similarly, in a PP-MS cohort from another study, CSF concentration of MBP significantly correlated with the prevalence of CSF CD8+CD20+ (*p* = 0.0005, r_s_ = 0.53), but not with CD4+CD20+ frequency or CSF total count of CD4+ or CD8+ T cells, suggesting a key role of CD8+CD20+ T cells in CNS demyelination, hence potentially identifying encephalitogenic T cell clones [32].

In a cohort of PP-MS patients, the percentage of CSF CD4+CD20+, but not of CD8+CD20+ memory, T cells showed a positive correlation with age (r = 0.6799, *p* = 0.0150), whereas the latter was inversely correlated with the frequency of CSF B cells (r = −0.6603, *p* = 0.0140) [33]. In addition to this, the observation that a relative abundance of CSF CD4+ compared with CD8+ memory T cells was associated with higher Age-Related Multiple Sclerosis Severity (ARMSS) scores led the authors to speculate that such expansion could be a contributor to progressive disease.

**Table 1:** Studies describing features of CD20+ T cells isolated from CSF and peripheral blood of MS patients and/or healthy donors.

Study [Ref]	Average Baseline Characteristics of the Population (MS/Controls or HD)	Findings in the Compartment Analyzed (Peripheral Blood and/or CSF)
Hultin et al., 1993 [4]	**HD:** 17.	**Peripheral blood:** Frequency of CD20+ T cells: 2.4 ± 1.5%; 83% CD3+, 12% CD56+, 8% CD19+, 6% HLA-DR. Expression of CD2, CD5, CD56, CD57, HLA-DR similar to CD20− T cells. ↓ CD4:CD8 ratio compared to the CD20− T compartment.
Storie et al., 1995 [21]	**HD:** 41, categorized as: elderly (n = 10, mean age: 76 y); adults (n = 18, mean age: 35 y) and cord blood (n = 13, full term normal deliveries).	**Peripheral blood:** Frequency of CD20+ T increasing with age: 0.3 ± 0.1% in the cord blood, 2.1 ± 1.1% in the 20–60 years-old group, 6.9 ± 3.2%. in the >60 year-old cohort. CD20dim expression restricted to 84 ± 11% CD8+ and 21 ± 9% of CD4 + T lymphocytes.
Henry et al., 2010 [20]	**HD:** 28; mean age: 44.6 y (± 8.3).	**Peripheral blood:** Mean frequency of CD20+CD3+ 2.2 (±0.4%), range 0.11–5.7%. No correlation with donors’ age.
Holley et al., 2014 [19]	**MS:** 11 RR- (91% F); age: 22–53 y; mean EDSS: 2.2; ≥1 relapse in the previous y. **HD:** 12 (92% F); age 21–51 y.	**Peripheral blood:** no significant difference between MS and HD in CD20+CD3+ frequency (2–6%) and CD4+CD20+:CD8+CD20+ ratio. ↑ proportion of CD20+ T cells secreted IFN-γ compared to CD20− ones (97.9% vs. 11.8%, respectively).
Palanichamy et al., 2014 [7]	**MS:** 11 untreated (64% F): 8 RR-, 2 CIS, 1 PP-; mean age: 44 y (range 25–61). **HD:** 18 (50% F); mean age: 36.8 (range 21–64).	**Peripheral blood:** Frequency of CD20+ T cells slightly higher in MS (7.2 ± 3.6% vs. 5.4 ± 2.4%) compared to HD in the whole CD3+ T cell pool. No significant differences between MS and HD for the T cell subsets naïve, T_CM_, T_EM_.
Schuh et al., 2016 [5]	**MS:** 6 untreated RR- during relapse. **Controls:** 8 OND without signs of CNS inflammation.	**CSF:** Similar frequency of CD3+CD20+ between groups. Predominance of CD20+ T compared to CD20+ B cells in OND; similar rate of CD20+ B and CD20+ T cells in MS.
Gingele et al., 2018 [17]	**MS:** 21 (62% F): 17 RR- and 4 PP-; median disease duration: 14.6 y and 5.6 y, respectively; free from prior DMTs (≥2 m) or not treated (respectively); mean age: 43 y (range 22–65).	**Peripheral blood:** CD20+ T cells constituted 18.4 ± 2.3% of all CD20+ cells. Larger expression of CD20+ on CD8+ than CD4+ T cells.
Von Essen et al., 2019 [13]	**MS:** -11 alemtuzumab-treated (2 years) RR-; mean age: 42 y (range 33–58); -25 untreated RR-, including 23 treatment-naïve, and 2 previously treated (IFN-β1a and teriflunomide; DMF); ≥1 m since last steroid treatment. Mean age: 36 y (range 22–52); -Replication cohort: 12 untreated PP-; mean age: 55. **HD:** 25; mean age: 37 y (range 24–56); -Replication cohort: 12 (mean age: 46).	**Peripheral blood:** ↑ frequency of CD20+ T cells and ↓ CD4+CD20+:CD8+CD20+ ratio in MS compared to HD. Features of high differentiation (less naïve and stem cell memory cells, more T_EM_ and terminal differentiated cells) and high intrinsic pathogenicity and CNS invasion potential (↑ expression of chemokine receptors CCR2, CCR5, CCR6, CXCR3, and adhesion molecules CD49dhi, MCAM-1 compared to CD20-CD3+ (both groups). **CSF:** Enrichment of CD20+ T cells compared to the blood. ↑ expression of CNS migration molecules (CCR2, CCR5, CCR6, MCAM-1) on CD20+ T compared to CD20− T cells in both MS and HD. Positive correlation between CSF CD20+CD3+ frequency and MBP levels and EDSS.
Quendt et al., 2021 [16]	**MS:** 15 untreated RR- (80% F). Mean disease duration: 3 (±1.2) y. Mean age at the time of diagnosis/blood sampling: 37 (±2.8)/39.3 (±2.8) y.	**Peripheral blood:** Frequency of CD20+:6.6 ± 0.6% in CD4+ and 18.8 ± 2.8% in CD8+ compartment. Highly differentiated and proinflammatory phenotypes effector T cells compared with CD20− T cells. ↑ CD49d and production of GM-CSF, IFN-γ, IL-17, TNF-α, and regulatory cytokines (IL-4, IL-10) compared to CD20-.
Ochs et al., 2022 [6]	**MS:** 11 untreated RR- from a cohort of 14 (57% F, mean age: 40.36 ± 9.09 y, median EDSS score: 3 ± 2.5, mean time since MS diagnosis: 11 ± 6.54 y, mean wash-out 8.15 ± 4.85).	**Peripheral blood:** CD4+CD20+ T cells exhibit a more activated CD69+ phenotype and ↑ CXCR3+ Th1 and CCR4+ Th2 cells frequency compared to CD4+CD20-. ↑ CD49d expression and ↑ frequencies of IFNγ, GM-CSF, and TNFα–producing cells in both CD4+ and CD8+CD3+. ↓ frequency of immature, naïve, and stem cell–like CD4+ and CD8+ memory cells, and ↑ frequencies of T_CM_ and T_EM_ cells compared to CD20− cells.
Von Essen et al., 2023 [32]	**MS:** 25 + 16 (validation cohort) untreated PP-.	**CSF:** Enrichment of CD20+ T compared to blood. Positive correlation between CSF CD8+CD20+ T cells prevalence and concentration of MBP (*p* = 0.0005; rs = 0.53).
Konen et al., 2024 [18]	**MS:** 53 relapsing (72% F); median age: 36 y (IQR: 29–46); median MS duration: 20 m (IQR: 2–84); median EDSS: 2.0 (IQR: 1–3.5).	**Peripheral blood:** T CD20+ represent 13% (±6%) of CD20+ cells, being composed by 54% (±20%) CD3+CD4+T helper cells and by 46% (±17%) CD3+CD8+cytotoxic T cells.
Oliveira Pinho et al., 2024 [22]	**HD:** 17 (76% F); mean age: 41 ± 10.8 y.	**Peripheral blood:** CD20+ T cells represent 4.44% ± 3.81 of total T cells, with particular enrichment in the CD8+ T compartment. ↑ proportion of T_EM_ and T_CM_ CD8+ T cells compared to CD20− T cells; ↑ frequency of CD45RO+ T cells in the central and T_EM_ compartments of CD4+ and CD8+ CD20+ T cells. ↑ expression of CCR5, CD25 and PD-1 and ↓ expression of TIM-3 compared to CD20− T cells; ↑ proportion of Th1/Th17 phenotype. ↑ production of TNF-α, IFN-γ, and IL-17; highest proportion of T cells producing simultaneously TNF-α, IFN-γ, and IL-17 among the CD20+ T cell compartment.
Van Puijfelik et al., 2024 [33]	**MS:** 13 untreated PP- (69% F); median age: 50 y; disease duration: 7.1 y; median EDSS: 4. Relapses/MRI activity the year before in 15.4% of cases.	**CSF:** ↑ proportion of CD4+ and CD8+ CD45RA- memory CD20+ T cells compared to peripheral blood, with ↑ CCR5 and CXCR3 production. Age correlated inversely with the frequency of B cells (r = −0.6603, *p* = 0.0140) and directly with the percentage of CD4+CD20+ (r = 0.6799, *p* = 0.0150), but not of CD8+CD20+ memory T cells.

Abbreviations: ↑, increase; ↓, reduction; CCS, corticosteroids; CD, cluster of differentiation; CCR, chemokine receptor; CIS, clinically isolated syndrome; CSF, cerebrospinal fluid; DMF, dimethyl-fumarate; DMT, disease-modifying therapies; EDSS, Expanded Disability Status Scale; F, female; GM-CSF, granulocyte-monocyte colony-stimulating factor; HD: healthy donor; IFN, interferon; IL, interleukin; IQR, interquartile range; m, months; MRI, magnetic resonance imaging; MS, multiple sclerosis; OND, other neurological diseases; PD-1, programmed-cell death protein 1; PP-, primary-progressive; RR-, relapsing-remitting; y, years; T_h_, T helper; TIM-3, T cell immunoglobulin and mucin domain-3; TNF, tumour necrosis factor.

## 6. Effect of DMTs on CD20+ T Cells

The effects of DMTs on CD20+ T cells are described in a few studies, overall showing a depletion of this population, although to a different extent according to the main mechanism of action and immunodepleting activity of each treatment. Most studies are focused on the use of CD20-depleting mAbs, whereas sparse evidence is available on other DMTs, hereafter clustered as sequestering (natalizumab, fingolimod), pulsed (alemtuzumab, cladribine), and platform (dimethyl-fumarate) therapies. Baseline characteristics of the populations included and main findings of each study are summarized in Table 2.

### 6.1. CD20-Depleting Monoclonal Antibodies

Amongst anti-CD20 mAbs, the chimeric RTX and humanized ocrelizumab (OCR) are the most widely used and studied in this area. In 21 MS patients (17 RR-, 4 PP-), peripheral blood sampled two weeks after the first loading dose (300 mg) of OCR showed a complete depletion of CD20-expressing cells (from 224.9 ± 24.6/μL to 0.57 ± 0.18/μL, *p* < 0.0001), significant also in the sub-group analysis of CD19+ and CD3+CD20+ [17]. Both RTX and OCR induce a complete depletion of B cells [16,34] with a consequent increase in the relative frequency of the remaining immune cells, except for a slight reduction in T cell frequency (14.5% vs. 17.3%) [16], especially in T_EM_ CD3+CD4+ (22.0%) and CD8+ cells, and a relative increase in the frequency of CD4+ and CD8+ naïve T cells [34]. This difference is mostly due to a complete depletion of CD20+ T cells in both the CD4+ and CD8+ T cell pools, which are significantly reduced compared to their CD20− T counterparts [16,34].

Over a longer follow-up after the RTX loading dose (two infusions of 1 g IV each, two weeks apart), a near-complete depletion of CD20+ T cells was described during weeks 1–12 (mean frequencies of 0.36 ± 0.36% vs. 7.8 ± 3.7% in untreated patients, *p* < 0.0001, with a major impact on CD8+ cells), followed by their partial repletion during weeks 25–36 (2.7 ± 2.3%) and 37–52 (2.8 ± 1.5%) [7]. Such depletion did not affect the overall CD3+ population, suggesting a rapid homeostatic repopulation with CD20− T cells [7]. After RTX infusion, CD3+ levels were significantly reduced by more than 50% even in the CSF, with a suggestive correlation with the decrease of CSF CXCL13, a chemoattractant factor for B cells and activated T cells, which could contribute to B cell depletion in this compartment [35]. Analyzing the expression of brain residency-associated T cell markers after one year of OCR treatment in PP-MS patients, van Puijfelik et al. reported a prominent loss of CXCR3+ and CCR5+ CD4+ and CD8+ T cell memory fractions compared to untreated PP-MS [33]. In the CSF, OCR-treated PP-MS exhibited a significant depletion of CCR5+, CXCR3+, CCR6+, and CCR4+ CD4+CD20+ memory T cells, suggesting a relation with the reduced proportion of their blood counterparts, but not in the CD8+ memory T cell pool [33].

Ofatumumab (OFA), a fully human anti-CD20 mAb, showed similar depletion and repletion kinetics between CD20+ B and CD20+ T cells over a 90-day follow-up in an animal model [36]. In a cohort of 53 MS patients treated with OFA, CD20+ lymphocytes were completely depleted after the first administration in all but three patients; at the same time point, CD3+CD20+ T lymphocytes were completely depleted in all the patients, while CD19+CD20+ B lymphocytes could still be measured at a low level in three patients [18]. After the second OFA administration, complete CD20+ (both CD3+ and CD19+) cell depletion in the peripheral blood was achieved in all MS patients, and this persisted after 3 months of OFA treatment, leading to a significant reduction of the absolute lymphocyte count in the peripheral blood [18].

The kinetics of immune cell repopulation after the administration of anti-CD20 mAbs suggest that CD20+CD3+ replenish earlier and at a higher frequency compared to B cells, probably due to an earlier repopulation of these latter in secondary lymphatic organs rather than in the blood; B cell activation may thereafter promote earlier migration of CD20+ T cells into the blood, inducing an inversion of the normally positive CD20+CD19+/CD20+CD3+ ratio. Conflicting evidence is available on the balance between anti- and pro-inflammatory phenotypes of the CD20+ T cell pool re-emerging after anti-CD20 mAb-mediated depletion. In the study by Ochs et al., CD20+ T cells seem to recur in a more pathogenic phenotype with trends towards more differentiation and higher expression of CD49d and pro-inflammatory cytokines (IFN-γ, GM-CSF, IL-17, TNFα) [6], similarly to previous descriptions of repopulated B cells [34,37]. On the contrary, Shinoda et al. highlighted a lower proinflammatory profile of the reemerging CD20+ T cell pool, showing reduced frequencies of T_EM_ cells and a minor expression of IFN-γ and TNF-α; a decreased ratio of proinflammatory to anti-inflammatory (IL-6/IL-10 and TNF-α/IL-10) cytokine-expressing B cells was also described [34]. Examining the antigen-specific response of T cells collected one to six months after anti-CD20 mAb (OCR or RTX) administration, no significant differences were found in the frequency of myelin- or influenza-specific CD8+ T cells, except for a small but significant reduction in influenza A M1_58–66_-specific CD8+ T cells; analyzing the CD20+CD8+ T cell subpopulation, the frequencies of T cells specific to myelin epitopes and the MOG_181–189_ individual epitope were reduced, consistent with preferential depletion of myelin-specific CD8+ T cells induced by the treatment [8].

Ublituximab, a third-generation glycoengineered chimeric anti-CD20 mAb [38], shows kinetics of B cell depletion and repopulation similar to those described for the other DMTs of the same class and promotes an anti-inflammatory shift in the T cell pool characterized by an increase in naïve CD8+ T cells at day 2 and an apparent decrease of effector and memory CD8+ T cells [39]. Although this could be due to a direct CD20+CD3+ depletion as evidenced in the CD8+ compartment, to our knowledge, no specific data on the effect of ublituximab on CD20+ T cells are available to date.

### 6.2. Sequestering DMTs

DMTs with a predominant mechanism of action based on preventing CNS invasion from activated lymphocytes include modulators of the sphingosine-1-phosphate receptor (“imod”) and natalizumab (NTZ).

Fingolimod (FTY), the first “imod” approved for MS, induces lymphocyte sequestering into secondary lymphoid organs through functional antagonism of the sphingosine-1-phosphate receptor [40]. Quendt et al. reported a reduction in the absolute count of CD20+ T cells in the peripheral blood during FTY treatment, impacting both the CD4+ and CD8+ subpopulations. Furthermore, FTY reduced the activation level of CD8+CD20+ T cells compared to baseline (decrease in CD69, CXCR3, and CCR6 expression), but it appeared to increase the relative frequency of differentiated CD20+ T cells, showing higher proportions of T_EM_ CD4+CD20+ and terminally differentiated CD3+CD20+ and lower proportions of naïve plus stem cell-like memory CD8+CD20+ and central memory (T_CM_) CD8+CD20+ cells compared to untreated MS [23]. However, no effect of FTY was observed on the frequency of CD20+ T cells secreting pro-inflammatory cytokines (GM-CSF, IFNγ, IL-17, and TNF-α) compared to untreated controls. To our knowledge, the impact of the remaining “imods” (ponesimod, ozanimod, and siponimod) on CD20+ T cells has not been described to date.

NTZ is an anti-alfa-4 integrin humanized mAb acting by preventing the transmigration of lymphocytes across the BBB, which is associated with a relative lymphocytosis in the peripheral blood. Opposite to findings with FTY treatment, NTZ induced a significant increase of CD8+CD20+ cell frequency in peripheral blood [16] and of the relative frequency and absolute number of CD20+ T cells when compared to untreated patients [5], with a minor effect on their phenotype consisting of a reduction of the relative expression of the adhesion molecule CD49d and activation marker CD69 on both CD4+ and CD8+ cells compared to untreated MS; again, no differences in pro-inflammatory cytokines secretion were observed compared to untreated MS, except for a reduction of IL-17 [16].

### 6.3. Pulsed Therapies

Pulsed therapies include alemtuzumab (ATZ) and cladribine (CLAD), sharing a similar schedule of administration (one/two yearly treatment cycles over a 2-year treatment course) and inducing transient immune cell depletion followed by repopulation, associated with a variable duration of disease remission in the absence of chronic DMT administration.

ATZ, an anti-CD52 mAb, reduced the absolute number of CD3+CD20+ when compared to untreated RR-MS patients (*p* < 0.01), without affecting their relative frequencies [5]. ATZ effectiveness on CD20+ T cells seems to persist beyond two years of treatment, as, despite partial recovery of the T cell pool, the percentage of CD20+ T cells was persistently reduced in a treated RR-MS cohort compared to untreated patients, with means of 1.99% vs. 7.73%, respectively (*p* < 0.0001), and this was associated with an increased CD4+/CD8+ CD20+ T cell ratio (*p* = 0.029) [13]. Similarly, 2 years after treatment, CD20+ cells were reduced in the CSF from ATZ-treated compared to untreated RR-MS patients, with a predominant treatment effect in the CD20+ T compared to the CD20+ B cell pool [13]. This effect might be due to a decreased expression of CD49dhi, CCR2, and CXCR3 (significant for CD8+CD20+ T cells only) in peripheral CD20+ T cells, likely compromising their ability to migrate to the CSF, although no changes in CCR5, CCR6, or MCAM-1 were observed [13].

CLAD is an oral therapy administered as a prodrug intracellularly activated by deoxycytidine kinase (DCK), which is highly expressed in adaptive immune cells; as a consequence, the active molecule accumulates preferentially within lymphocytes, where it impairs DNA synthesis and induces cell death [41,42]. Moser et al. reported a significant expansion of the CD20+ T cell subsets at month 21 after treatment with CLAD (+129% compared to baseline), but no further phenotypic characterization was provided [43].

### 6.4. Platform DMTs

Dimethyl-fumarate (DMF), an oral fumaric acid ester acting on many intracellular targets within adaptive immune cells, besides reducing the overall frequency of T cells [32,44], decreases the absolute number of blood CD3+CD20+ cells in RR-MS compared to no-treatment, without affecting their relative frequencies [5]. Within the CD3+ pool, DMF seems to preferentially affect CD20+ T cells, especially CD8+CD20+, with a marginal but significant reduction of CD4+CD20+ compared to their CD20− counterparts [13]. Similarly to NTZ, DMF modulated some encephalitogenic features of CD20+ T cells, as evidenced by a reduction of their level of differentiation (decrease in T_EM_ CD4+CD20+ cells), activation (reduction of CXCR3 and CCR6), production of pro-inflammatory cytokines (GM-CSF and IL-17, but also IFN-γ and TNF-α), and expression of the adhesion molecule CD49d [13].

A significant reduction of frequency and absolute number of peripheral CD4+ and CD8+CD20+ cells in peripheral blood was confirmed by von Essen et al. in PP-MS patients treated with DMF for 48 weeks [32]. However, the absolute number of CD4+CD20+ cells in the CSF was unaffected by DMF, despite a decrease in the overall CSF T cell population, this latter likely a consequence of the reduction in peripheral circulating T cells. Surprisingly, the relative frequency of CD4+CD20+ and the absolute number and relative frequency of CD8+CD20+ T cells in the CSF were even higher in DMF- than in placebo-treated MS [32]. Although long-term treatment with DMF could affect the intrathecal CD20+ T cell pool as a consequence of diminished renewal from the peripheral blood, these findings suggest that DMF does not affect intrathecal CD20+ T cells, supporting the hypothesis that these could be part of the CNS compartmentalized immune response, as supported by anatomopathological evidence of CD20+ T cells with a T_RM_ phenotype [31].

No data are available, to our knowledge, on the effect of the remaining platform DMTs (teriflunomide, interferons, and glatiramer acetate) on CD20+ T cells.

**Table 2:** Studies describing the effect of available disease-modifying treatments on CD20+ T cells in patients with MS.

Ref.	DMT/Control Group ^a^	Timepoint ^b^	Effect on CD20+ T Cells Isolated from Peripheral Blood and/or CSF
Piccio et al., 2010 [35]	**Rituximab:** 30 RR-MS (73% F); four weekly doses (375 mg/m^2^ each).	−1 w, +24–30 w	**Peripheral blood:** ↓ CD3+ T cells (−12%); ↓ CXCL13, CCL19. **CSF:** ↓ CD3+ levels (>50%) with a suggestive correlation with the decrease of CSF CXCL13.
Palanichamy et al., 2014 [7]	**Rituximab**: 11 untreated MS (64% F): 1 PP-, 2 CIS, 8 RR-; age: 44 y (25–61). Two IV infusions (1 g each) two weeks apart. **Control Group**: 18 HD (50% F), age 36 y (21–64).	+1–12 w (n = 7), +13–24 w (n = 8), +25–36 w (n = 5), +37–52 w (n = 5)	**Peripheral blood:** near-complete depletion of CD20+CD3+ during w 1–12 (mean frequencies 0.36 ± 0.36% vs. 7.8 ± 3.7% in untreated; major impact on CD8+), partial repletion during w 25–36 (2.7 ± 2.3%) and w 37–52 (2.8 ± 1.5%).
Schuh et al., 2016 [5]	**Alemtuzumab:** 4 RR-MS (50% F), age: 24.5 y (24–37). **Natalizumab:** 9 RR-MS (56% F), age: 46 y (25–61). **Fingolimod:** 6 RR-MS (67% F), age: 44.5 y (29–62). **Dimethyl-fumarate:** 9 RR-MS (44% F), age: 46 y (33–60). **Control Group:** 10 untreated RR-MS (70% F), age: 40.5 y (20–67).	N.R.	**Peripheral blood:** Alemtuzumab, Fingolimod and Dimethyl-fumarate: ↓ absolute number of CD20+ T cells, but not of their relative frequency vs. controls. Natalizumab: ↑ relative frequency and absolute number (*p* < 0.0001) of CD20+ T cells vs. controls.
Gingele et al., 2018 [17]	**Ocrelizumab:** 21 MS (62% F), age: 43 y (22–65); 17 RR- and 9 PP- (disease duration: 14.6 y and 5.6 y, respectively).	Pre-treatment, +2 w (before 2nd dose)	**Peripheral blood:** Complete depletion of CD20+ cells (from 224.9 ± 24.6/μL to 0.57 ± 0.18/μL), confirmed in both the CD19+ and CD3+CD20+ populations.
Lovett-Racke et al., 2019 [39]	**Ublituximab:** 47 RR-MS (65% F); disease duration: 7.4 y (18–55); EDSS ≤ 5.5; ≥2 relapses in prior 2 years or 1 relapse in the past year and/or ≥ 1 Gd+ lesion, stability ≥30 d.	w 0 (pre-infusion), +2 d, +2 w, +3 w, +every 4 w up to w 24	**Peripheral blood:** Change in the ratio of naïve to memory T cells in the CD8+ compartment on +2 d; ↓ percentage of Th1; ↑ Treg frequency.
Sabatino et al., 2019 [8]	**Rituximab or Ocrelizumab:** 9 RR-MS.	Baseline, +1–6 m after latest infusion	**Peripheral blood:** No significant difference in frequencies of myelin- and influenza-specific CD8+ T cells; ↓ myelin-specific epitopes and MOG_181–189_ individual epitope specific-T cell frequency in CD20+CD8+ subpopulation.
Von Essen et al., 2019 [13]	**Alemtuzumab:** 11 RR-MS; age: 42 y (33–58). **Control Group:** 25 untreated RR-MS; age: 36 y (22–52).	+2 y	**Peripheral blood:** ↓ CD20+ T cells vs. controls (1.99% vs. 7.73%), with a trend for ↑ CD4/CD8 CD20+ T cells ratio compared to untreated; ↓ expression of CD49dhi, CCR2 and CXCR3 in CD20+ T cells. **CSF:** ↓ CSF CD20+ T cell pool in ATZ cohort, with greater relative ↓ of CD20+ T cells compared to CD20+ B cells within total CSF lymphocytes.
Quendt et al., 2021 [16]	**Natalizumab:** 16 RR-MS (56% F); disease duration/age at diagnosis: 4.8 (±1.1) y/28.7 (±2.3) y. **Fingolimod:** 22 RR-MS (50% F); disease duration/age at diagnosis: 9 (±1.3) y/37 (±2.8) y. **Dimethyl-fumarate:** 20 RR-MS (50% F); disease duration/age at diagnosis: 4.3 (±0.9) y/33.3 (±2.3) y. **Rituximab or ocrelizumab:** 10 RR-MS (60% F); disease duration/age at diagnosis: 8.6 (±1.5 y)/40.2 (±5.7) y. **Control Group:** 15 untreated RR-MS (80% F); disease duration/age at diagnosis: 3 (±1.2) y/37 (±2.8) y.	N.R.	**Peripheral blood:** Natalizumab: ↑ in CD8+CD20+ T cell frequency; Relative ↓ expression of adhesion molecules (CD49d) and activation marker CD69. Fingolimod: ↓ CD8+CD20+ frequency, slighter ↓ of CD4+CD20+ frequency; ↓ activation levels of CD8+CD20+ cells (↓ CD69, CXCR3, CCR6); ↑ relative frequency of differentiated CD20+ T cells; No significant difference in CD49d expression and in GM-CSF, IL-17, IFN-γ, and TNF-α CD20+ T cells production. Dimethyl-fumarate: ↓ CD8+CD20+ frequency, no significant difference for CD4+CD20+; ↓ differentiation level (↓ in T_EM_ CD4+CD20+ cells, but ↑ naïve and stem cells-like memory CD4+CD20+); ↓ activation (CXCR3, CCR6, Cd69, CD49d); ↓ IL-17, GM-CSF, IFN-γ, and TNF-α CD4+CD20+ producing cells. Rituximab or ocrelizumab: complete eradication of CD20+ T cells.
Ochs et al., 2022 [6]	**Rituximab:** 14 MS (57% F); EDSS 3 (±2.5); age: 40.36 (±9.09) y; disease duration: 11 (±6.54) y; wash out from previous treatment (3 DMT, 4 FTY, 1 azathioprine, 1 NTZ, 1 glatiramer acetate, 3 CCS, 1 naïve): 8.15 (±4.85) w.	Baseline, +0–3 m, +3–14 m	**Peripheral blood:** Depletion of both CD20+ B and T cells; earlier reappearance of CD20+ T than B cells in the blood; On an individual basis, ↑ frequency of CD49dhi cells and proinflammatory cytokines IFNγ, GM-CSF, TNFα, and IL-17–producing cells.
Von Essen et al., 2023 [32]	**Dimethyl-fumarate:** -blood: 21 PP-MS; -CSF: 15 PP-MS. **Control Group:** -Blood: 24 placebo-treated PP-MS; -CSF: 14 placebo-treated PP-MS.	Baseline, +48 w	**Peripheral blood:** ↓ frequency and absolute number of CD4+ and CD8+CD20+ cells. **CSF:** ↑ absolute number and relative frequency of CD8+CD20+, ↑ relative frequency of CD4+CD20+.
Shinoda et al., 2023 [34]	**Ocrelizumab:** -Discovery cohort: 23 MS (13 RR-, 10 SP-/PP-) (52.2% F); age: 48.2 (±13.3) y; EDSS: 2.7 (±1.8); treatment-naïve: 100%. -Validation cohort: 35 RR-MS (60% F); age: 37.3 (±10.3) y; EDSS: 2.1 (±1.2); treatment-naïve: 54.3%.	Baseline, +2–4 m	**Peripheral blood:** inverse association between baseline frequency of circulating CD20+ T cells (particularly CD8+) and with Gd+ lesions; no correlation with disease activity during treatment; ↓ frequencies of T T_EM_ and ↓ expression of the proinflammatory cytokines IFN-γ and TNF-α in the reemerging CD20+CD8+ T cell pool.
Konen et al., 2024 [18]	**Ofatumumab:** 53 relapsing MS (72% F); age: 36 y (29–46); MS duration: 20 m (2–84); EDSS: 2.0 (1–3.5).	Pre-treatment, +1 w, +2 w, +3 m	**Peripheral blood:** Complete depletion of CD3+CD20+ T cells after the first administration in all the patients, persisting after 3 m of treatment.
Moser et al., 2024 [43]	**Cladribine:** 18 MS (83% F); age: 36.4 (±11.7) y; disease duration: 8.8 (0–25) y; EDSS 2.25 (±1.7).	Every 3 months from baseline to +2 y	**Peripheral blood:** ↑ frequency of CD20+ T cells (+127% at m 21).
Van Puijfelik et al., 2024 [33]	**Ocrelizumab:** 12 PP-MS (58% F); age: 57.6 y; disease duration: 8.1 y; EDSS 4.25; 8.3% had a relapse and 50% had MRI activity during the previous y. **Control Group:** 13 untreated PP-MS (69% F); age: 50 y; disease duration: 7.1 y; EDSS: 4; 15.4% had relapses and MRI activity the previous y.	+ median 117 d (14–189) after latest OCR dose in the treatment group (except for 3 cases: +33, 35, or 38 w)	**Peripheral blood:** ↓ CXCR3+ and CCR5+ T cell memory fractions compared to the control group. **CSF:** ↓ CCR5+, CXCR3+, CCR6+, and CCR4+ CD4+CD20+ memory T cells in treated vs. untreated patients.

^a^ Characteristics of the patient/control population are provided as average values (median/mean) and related dispersion measure (range, confidence interval, or standard deviation), as provided by the source publication. ^b^ Timepoint with respect to treatment with the analyzed DMT. Abbreviations: ↑, increase; ↓, reduction; CCS, corticosteroids; CD, cluster of differentiation; CCR, chemokine receptor; CIS, clinically isolated syndrome; CSF, cerebrospinal fluid; d, days; DMF, dimethyl-fumarate; DMT, disease-modifying therapies; EDSS, Expanded Disability Status Scale; F, female; Gd+, gadolinium-enhancing; GM-CSF, granulocyte-monocyte colony-stimulating factor; HD: healthy donor; IFN, interferon; IL, interleukin; IQR, interquartile range; m, months; MRI, magnetic resonance imaging; MS, multiple sclerosis; N.R., not reported; NTZ, natalizumab; OCR, ocrelizumab; OND, other neurological diseases; PD-1, programmed-cell death protein 1; PP-, primary-progressive; RR-, relapsing-remitting; SP-, secondary progressive; y, years; T_EM,_ T effector memory; Th, T helper; TNF, tumor necrosis factor; w, weeks.

## 7. CD20+ T Cells as Potential Biomarkers of Disease Activity

Few data are available so far on the role of CD20+ T cells as potential biomarkers of disease activity in MS, highlighting potential associations between this cell population and relapses, MRI activity, and serum/CSF markers of demyelination and axonal damage.

A potential association between early repopulation of CD20+ T cells and MS relapse was suggested by Schuh et al., reporting that three out of eight MS patients treated with RTX relapsed 4–8 months after the last infusion, at a time when more CD20+ T cells than B cells were present, as they seem to repopulate earlier than B cells in the blood [5]. Interestingly, in one patient, a relapse occurred when CD20+ B cells were barely detectable in the blood, whereas CD20+ T cells had reached 4% of all lymphocytes [5]. At first sight, this evidence seems conflicting with the observation, in an untreated MS cohort undergoing anti-CD20 mAb treatment, of an inverse correlation between both CD20+CD8+ and brain MRI gadolinium-enhancing lesions before treatment, and between baseline CD20+CD8+ levels and new brain lesions developing from week 12 following treatment start [34]. However, this apparent paradox could be solved by considering that CD20+ T cells might migrate out of the circulation to participate in the early development of new disease activity in one or more disease-relevant compartments [34]. Aligned with this hypothesis, a positive correlation was reported by Von Essen et al. between the percentage of CSF CD8+ CD20+ cells at baseline and the number of new T2 lesions after 48 weeks of DMF treatment in PP-MS [32]. Accordingly, in CSF from a PP-MS cohort, a positive correlation between CD8+CD20+ and WM lesion volume (*p* = 0.0092; r_s_ = 0.43; not for CD4+CD20+, *p* = 0.27; r_s_ = 0.19) and a negative correlation between NAWM and thalamus volume with CD8+CD20+ (*p* = 0.021, r_s_ = −0.39; *p* = 0.0058; r_s_ = −0.46, respectively) and CD4+CD8+ (*p* = 0.044; r_s_ = −0.34; *p* = 0.0041, r_s_ = −0.48, respectively) cells were described; no correlations were observed with cortical GM volume, magnetization transfer in WM lesions, or fractional anisotropy in NAWM [32].

Breakdown of myelin followed by release of its proteins, including MBP, in the CSF is a hallmark of MS [45]. A positive correlation between the percentage of CD20+ T cells in the CSF of RR-MS patients and levels of MBP and a suggestive correlation with EDSS were described, without any correlations with white blood cell count, IgG index, or percentage of B cells in the CSF [13]. The correlation between CSF concentration of MBP and CSF prevalence of the CD8+CD20+ subpopulation was confirmed in a PP-MS cohort (*p* = 0.0005; r_s_ = 0.53), where no significant correlations were observed with CD4+CD20+ (*p* = 0.51; r_s_ = 0.11), total CD4+ (*p* = 0.12; r_s_ = 0.25), and CD8+ T cells (*p* = 0.13; r_s_ = 0.25), nor between CSF neurofilament light chains (NFL) concentrations and CD4+CD20+ (*p* = 0.33; r_s_ = 0.17) or CD8+CD20+ frequencies (*p* = 0.057; r_s_ = 0.32) [32].

## 8. Conclusions

CD20+ T lymphocytes represent a distinct T cell population characterized by a pro-inflammatory phenotype, a robust response to myelin antigens, and an increased potential for CNS migration compared to their CD20− counterpart. Evidence from preclinical and clinical studies supports their active involvement in MS pathogenesis, strengthening the concept of MS as a predominantly T cell-mediated disease and paving the way for future development of CD20+ T cells as a biomarker of MS activity and treatment response. Amongst currently available treatments, CD20-depleting mAbs deeply affect CD20+ T cells, whereas few data are available on the remaining DMTs, overall suggesting a modest impact on CD20+ T cell frequency and activation. Targeted depletion of CD20+ T cells could represent a promising strategy for future DMT development. In this respect, CD19/CD20 bispecific CAR-T cells were recently successfully expanded from MS patients and HD and demonstrated to selectively deplete CD20+ T cells in culture [46]. Moreover, the presence of CD20+ T cells has been observed in other autoimmune conditions, representing a new avenue of study into their broader role in immune-mediated diseases.

## Data Availability

No original data were presented in this article.

## Abbreviations

ATZ	Alemtuzumab
CCS	Corticosteroids
CD	Cluster of differentiation
CCR	Chemokine receptor
CIS	Clinically isolated syndrome
CLAD	Cladribine
CSF	Cerebrospinal fluid
DMF	dimethyl-fumarate
DMT	Disease-modifying therapies
EDSS	Expanded Disability Status Scale
FTY	Fingolimod
GM-CSF	Granulocyte-monocyte colony-stimulating factor
HD	Healthy donor
IFN	Interferon
IL	Interleukin
MRI	Magnetic resonance imaging
MS	Multiple sclerosis
NAWM	Normal appearing white matter
NTZ	Natalizumab
OCR	Ocrelizumab
OFA	Ofatumumab
OND	Other neurological diseases
PD-1	Programmed-cell death protein 1
PP-	Primary-progressive
RTX	Rituximab
RR-	Relapsing-remitting
T_CM_	T central memory
T_EM,_	T effector memory
T_h_	T helper
TIM-3	T cell immunoglobulin and mucin domain-3
TNF	Tumour necrosis factor
T_RM_	Tissue resident memory
WM	White matter
